# Unhealthy lifestyles and brain condition: Examining the relations of BMI, living alone, alcohol intake, short sleep, smoking, and lack of exercise with gray matter volume

**DOI:** 10.1371/journal.pone.0255285

**Published:** 2021-07-30

**Authors:** Keisuke Kokubun, Juan Cesar D. Pineda, Yoshinori Yamakawa

**Affiliations:** 1 Open Innovation Institute, Kyoto University, Kyoto, Japan; 2 Smart-Aging Research Center, Tohoku University, Sendai, Japan; 3 ImPACT Program of Council for Science, Technology and Innovation (Cabinet Office, Government of Japan), Chiyoda, Tokyo, Japan; 4 Institute of Innovative Research, Tokyo Institute of Technology, Meguro, Tokyo, Japan; 5 Office for Academic and Industrial Innovation, Kobe University, Kobe, Japan; 6 Brain Impact, Kyoto, Japan; Sao Paulo State University (UNESP), BRAZIL

## Abstract

Unhealthy lifestyles are damaging to the brain. Previous studies have indicated that body mass index (BMI), alcohol intake, short sleep, smoking, and lack of exercise are negatively associated with gray matter volume (GMV). Living alone has also been found to be related to GMV through lowered subjective happiness. However, to our knowledge, no GMV study has dealt with these unhealthy lifestyles simultaneously. By our analyses based on 142 healthy Japanese participants, BMI, alcohol intake, living alone, and short sleep were negatively associated with the gray-matter brain healthcare quotient (GM-BHQ), an MRI-based normalized GMV, after controlling for age, sex, and facility, not only individually but also when they were entered into a single regression model. Moreover, there were small but significant differences in the proportion of the variance for GM-BHQ explained by variables in a regression model (measured by R squared) between when these unhealthy variables were entered in an equation at the same time and when they were entered separately, with the former larger than the latter. However, smoking and lack of exercise were not significantly associated with GM-BHQ. Results indicate that some kinds of unhealthy lifestyles are somewhat harmful on their own, but may become more noxious to brain condition if practiced simultaneously, although its difference may not be large. To our knowledge, this study is the first to show that overlapping unhealthy lifestyles affects the brains of healthy adults.

## Introduction

Lifestyle factors can be potent in determining both physical and mental health. In modern affluent societies, the diseases leading to mortality—such as cardiovascular disorders, obesity, diabetes, and cancer—are now strongly determined by lifestyle [1, 2]. It is indicated in the previous research that differences in just four lifestyle factors—smoking, physical activity, alcohol intake, and diet—exert a major impact on mortality, and even small differences in lifestyle can make a major difference in health status [3, 4]. These findings point to the importance of a comprehensive modulation of lifestyle factors when finding ways to preserve good conditions of health. Similarly, another study revealed that a healthy lifestyle, assessed by a simple composite index composed of physical exercise, dietary habits, BMI, smoking and alcohol consumption, is related to better memory performance in healthy elderly individuals [5], which suggests that certain lifestyles may be better at enhancing brain volume or delaying brain atrophy [6].

In the area of brain science, many studies show negative correlations between BMI and regional gray matter volumes, although the regional patterns often varied between studies [7–9]. Recently, using a large population-based cohort of 617 healthy older adults, a higher BMI correlated significantly with lower GMV in multiple brain regions, including (pre)frontal, temporal, insular and occipital cortex, thalamus, putamen, amygdala, and cerebellum [10]. In line with this finding, a study on 144 Japanese healthy adults found that whole-brain normalized GMV is also negatively correlated with BMI [11].

Subpar sleep quantity and quality are also shown to be harmful to the brain. In a case-control study, medication-free chronic primary insomnia patients had smaller GMV in the left orbitofrontal cortex, and anterior and posterior precuneus [12]. In the same vein, low sleep quality (the number of nocturnal awakenings) was negatively correlated with GMV in the insular region in late adulthood [13]. Likewise, reduced GMV was correlated with increased daytime sleepiness in the elderly [14]. These are studies of people with insomnia and the elderly. However, even in studies on adolescents, the GMV of medial prefrontal—anterior cingulate cortex correlated inversely with both weekend bedtime and wake-up time, and also with poor school performance [15].

Alcohol consumption is inversely correlated with GMV. A longitudinal study compared 35 heavy drinking adolescents with no alcohol use disorder and 27 light drinking controls. GMVs were significantly smaller among heavy drinking participants in the bilateral anterior cingulate cortex, right orbitofrontal and frontopolar cortex, right superior temporal gyrus, and right insular cortex compared to the control group [16]. Furthermore, several studies have reported that the degree of brain atrophy is correlated with the rate and amount of alcohol consumed over a lifetime [17, 18]. To verify the findings in the current literature, a study on 171 healthy adults suggested that higher alcoholic intake is correlated with lower whole brain GMV [19]. Similarly, previous studies have indicated that smoking [20–22] and lack of exercise [23–25] are negatively associated with GMV.

In Japan, along with other wealthy countries, the preference for a one-person household has become a norm. This solo lifestyle, or living alone, is found to be detrimental to overall health [26–28] and psychological well-being [29]. Data from the Japanese General Social Survey from 2000 to 2010 indicate that young adults who live alone are significantly less happy than those who live with others [30]. Although not directly related to living alone, previous brain research found a positive relationship between the subjective happiness score and GMV in the right precuneus [31] and gray matter density of the rostral anterior cingulate cortex (rACC) [32], which opens up the idea that living alone may also have negative effects on GMV. However, as far as the direct relationship between living alone and GMV is concerned, there seem to be no published studies yet, to the best of our knowledge.

Current literature in the brain sciences shows that these unhealthy lifestyles are independently associated with GMV. However, to date, no studies have investigated GMV with these unhealthy behaviors combined. Also, the relationship between living arrangements and GMV (which we posit to have a negative relationship) has not yet been studied. Investigating GMV with obesity, living arrangements, alcohol consumption, sleeping habits, smoking, and exercises, combined, will, therefore, be the significant contribution of this study.

This study will focus on the relationship between GMV and the six unhealthy behaviors (obesity, living alone, alcohol intake, short sleep, smoking, and lack of exercise) individually and in combination. To quantify the GMV, we used a brain health measuring tool, called the gray-matter brain healthcare quotient (GM-BHQ: ITU-T, 2018). Based on previous studies and experiments, the GM-BHQ is found to be inversely correlated with age, BMI [11], stress, fatigue [33], personalities [34], and alcohol and animal food intake [19]. The GM-BHQ is derived as an average of standardized gray matter measures for 116 brain regions based on the automated anatomical labeling atlas [35]. Since this measure reflects GMV, we hypothesized that it could vary based on unhealthy lifestyles. We are led to the following six hypotheses.

H1: BMI is negatively related to GM-BHQ.
H2: Living alone is negatively related to GM-BHQ.
H3: Alcohol intake is negatively related to GM-BHQ.
H4: Short sleep is negatively related to GM-BHQ.
H5: Smoking is negatively related to GM-BHQ.
H6: Lack of exercise is negatively related to GM-BHQ.

Therefore, in this study, we investigated the cross-sectional relationship between the GM-BHQ of healthy participants and the results of a questionnaire about lifestyles.

## Materials and methods

### Subjects

One hundred and forty-two healthy participants (79 females and 63 males), aged 23–69 (mean (M) ± standard deviation (SD): 49.7 ± 10.2 years) were recruited in Kyoto and Tokyo (88 and 54 subjects, respectively), Japan, in May to June 2017. Table 1 indicates the female/male ratio for each location. Prospective subjects with any record of neurological, psychiatric, or other medical conditions that may impact the central nervous system were not recruited. In addition, no participant was excluded after the initial screening. This study was approved by the ethics committees of Kyoto University (approval number 27-P-13) and Tokyo Institute of Technology (approval number A16038) and was performed in accordance with the guidelines and regulations of these research institutions. All participants gave written informed consent before participation, and participant anonymity was preserved.

**Table 1:** Female/male ratio for each location.

	Female	Male	Total
Kyoto	42	46	88
	(47.7%)	(52.3%)	(100.0%)
Tokyo	37	17	54
	(68.5%)	(31.5%)	(100.0%)
Total	79	63	142
	(55.6%)	(44.4%)	(100.0%)

### Unhealthy lifestyle scales

We created new questionnaires to quantify living alone, alcohol intake, short sleep, smoking, and lack of exercise. Living alone was dichotomously measured by 1 (live alone) or 0 (live with somebody). For alcohol intake, we asked frequency from 1 (no intake), 2 (once for a month), 3 (2 to 3 times for a month or once for a week), 4 (2 to 3 times for a week), and 5 (more than 4 times for a week). For short sleep, the choices were 1 (more than 9 hours), 2 (8 hours), 3 (7 hours), 4 (6 hours), and 5 (less than 5 hours). Smoking was dichotomously measured by 1 (smoking) or 0 (non-smoking). For lack of exercise, we asked frequency from 1 (more than 2 times for a week), 2 (once for a week), 3 (1 to 2 times for a month), and 4 (no exercise). BMI was calculated using the standard formula: weight (kg) / [height (m)]^2^.

### MRI data acquisition

All magnetic resonance imaging (MRI) data were collected using a 3-T Siemens scanner (Verio, Siemens Medical Solutions, Erlangen, Germany or MAGNETOM Prisma, Siemens, Munich, Germany) at Kyoto University and 3-T General Electric scanner (Signa, GE Healthcare, Massachusetts, United States) at Tokyo Institute of Technology with a 32-channel head array coil. A high-resolution structural image was acquired using a three-dimensional (3D) T1-weighted magnetization-prepared rapid-acquisition gradient echo (MP-RAGE) pulse sequence. The parameters were as follows: repetition time (TR), 1900 ms; echo time (TE), 2.52 ms; inversion time (TI), 900 ms; flip angle, 9°; matrix size, 256 × 256; field of view (FOV), 256 mm; and slice thickness, 1 mm.

### MRI data analysis

The calculation of the GM-BHQ in this study is identical to the calculation methods used in a previous neuroimaging study [11]. In summary, gray matter images were segmented from T1-weighted images using Statistical Parametric Mapping 12 (SPM12; Wellcome Trust Centre for Neuroimaging, London, UK) running on MATLAB R2015b (Mathworks Inc., Sherborn, MA, USA), followed by spatial normalization using diffeomorphic anatomical registration through an exponentiated lie algebra (DARTEL) algorithm [36] and modulation to preserve the GM volume. All normalized, segmented, and modulated images were smoothed with an 8-mm full width at half-maximum (FWHM) Gaussian kernel. Additionally, intracranial volume (ICV) was calculated by summing the GM, white matter, and cerebrospinal fluid images for each subject. Proportional GM images were generated by dividing smoothed GM images by ICV to control for differences in whole-brain volume across participants. Using these proportional GM images, images for the mean and standard deviation (SD) across participants were generated. Then, we calculated the GM-BHQ using the following formula: 100 + 15 × (individual proportional GM—mean) / SD. Regional GM quotients were then extracted using an automated anatomical labeling (AAL) atlas [35] and averaged across regions to produce participant-specific GM-BHQ.

### Statistical analysis

To investigate the correlation between the GM-BHQ and various variables, we employed hierarchical regression analysis. We entered the control variables of age, sex, and facility in Step 1 and the main variables of unhealthy lifestyles individually in Step 2, respectively. We designated one for one facility and zero for another facility as done by prior multi‐facility data analysis [37, 38], which indicated controlling for a facility is a sufficient statistical technique in this situation. In step 3, we entered all the variables that were significant in Step 2. We added these respective variables to the models based on the hypothesis that variables of unhealthy lifestyles are closely related to GM-BHQ after adjusting for age, sex, and facility. The significance level was set at p < 0.05. We measured the fit of the regression model by R-squared (R^2^), the proportion of the variance for a dependent variable that’s explained by independent variables in a regression model. We determined whether the change in R^2^ (ΔR^2^) is statistically significant by F-test where a significant F-change means that the variables added in that step significantly improved the prediction [39]. Moreover, we determined whether theΔR^2^ is large enough by the “effect size” criterion where it is “small” for 0.02, “moderate” for 0.13, and “large” for 0.26 for R^2^ [40]. All statistical analyses were performed using IBM SPSS Statistics Version 26 (IBM Corp., Armonk, NY, USA).

## Results

Descriptive statistics of subjects and correlation coefficients between variables are shown in Table 2. GM-BHQ correlated with age (r = -0.667, p < 0.001), sex (r = 0.449, p < 0.001), and facility (r = -0.518, p < 0.001). The significant correlation between GM-BHQ and facility is most probably due to the difference of MRI machines used for the experiments. Moreover, GM-BHQ negatively correlated with BMI (r = -0.435, p < 0.001) and alcohol intake (r = -0.241, p < 0.01). However, GM-BHQ did not correlate with living alone (r = 0.057, p > 0.05), short sleep (r = 0.107, p > 0.05), smoking (r = -0.117, p > 0.05), and lack of exercise (r = 0.081, p > 0.05). Moreover, no pair among the six unhealthy life style variables showed significant association with each other, indicating that unhealthy behaviors are, in most cases, practiced by different persons in different magnitudes (r: correlation coefficient).

**Table 2:** Descriptive statistics and correlations.

Variable	Mean	SD	1	2	3	4	5	6	7	8	9
1. GM-BHQ	99.423	8.470									
2. Age	49.732	10.183	-0.667***								
3. Sex (male = 1, female = 2)	1.556	0.499	0.449***	-0.163							
4. Facility	0.620	0.487	-0.518***	0.408***	-0.203*						
5. BMI	22.832	3.322	-0.435***	0.229**	-0.388**	0.208*					
6. Living alone	0.176	0.382	0.057	-0.228**	0.152	-0.133	-0.014				
7. Alcohol intake	3.092	1.473	-0.241**	0.082	-0.263**	-0.020	0.122	0.022			
8. Short sleep	3.732	0.922	0.107	-0.142	0.249**	-0.165*	-0.068	-0.046	-0.081		
9. Smoking	0.077	0.268	-0.117	0.057	-0.112	0.010	0.003	0.004	0.161	-0.002	
10. Lack of exercise	2.599	1.179	0.081	-0.122	0.153	0.140	-0.078	-0.047	-0.077	-0.054	0.009

n = 142

*p < 0.05

**p < 0.01

***p < 0.001.

Living alone: 1 for “live alone”; and 0 for “live with somebody”.

Alcohol intake: 1 for “no intake”; 2 for “once for a month”; 3 for “2 to 3 times for a month or once for a week”; 4 for “2 to 3 times for a week”; and 5 for “more than 4 times for a week”.

Short sleep: 1 for “more than 9 hours”; 2 for “8 hours”; 3 for “7 hours”; 4 for “6 hours”; and 5 for “less than 5 hours”.

Smoking: 1 for “smoking”; and 0 for “non-smoking”.

Lack of exercise: 1 for “more than 2 times for a week”; 2 for “once for a week”; 3 for “1 to 2 times for a month”; and 4 for “no exercise”.

Table 3 shows the results of regression analyses. In Step 1, age (R = 0.782, β = -0.516, p < 0.001), sex (R = 0.782, β = 0.315, p < 0.001), and facility (R = 0.782, β = -0.244, p < 0.001) were significant, indicating that GM-BHQ scores tended to be lower in the male and elderly than the female and younger participants after controlling for the difference of the used MRI machine. In Step 2, BMI (R = 0.798, β = -0.176, p = 0.002), live alone (R = 0.796, β = -0.152, p = 0.005), and alcoholic intake (R = 0.792, β = -0.132, p = 0.017) were independently significant after controlling for sex, age, and facility. These results indicate that GM-BHQ scores are negatively associated with these unhealthy life styles, individually. However, short sleep (R = 0.787, β = -0.092, p = 0.096), smoking (R = 0.784, β = -0.051, p = 0.346), and lack of exercise (R = 0.782, β = 0.004, p = 0.938) were not significant. Moreover, ΔR2 from Step 1 to Step 2, which was 0.025 for BMI and for 0.021 for living alone, were larger than 0.02, an effect size that is “small”, but smaller than 0.13, an effect size that is “moderate”, by Cohen’s criterion [40], indicating that these two unhealthy lifestyle variables had small but significant relationships with GM-BHQ. However, for other variables, increments of R^2^ from Step 1 to Step 2 were 0.016 for alcohol intake, 0.008 for short sleep, 0.003 for smoking, and 0.000 for lack of exercise, indicating marginal or almost no effects to GM-BHQ (R: multiple correlation coefficients; β: regression coefficient; R^2^: coefficient of determination).

**Table 3:** Multiple regression analysis of unhealthy lifestyle factors on GM-BHQ.

	GM-BHQ															
	Step 1		Step 2												Step 3	
	β^a, b^	p-value	β^a, b^	p-value	β^a, b^	p-value	β^a, b^	p-value	β^a, b^	p-value	β^a, b^	p-value	β^a, b^	p-value	β^a, b^	p-value
**Control variables**																
Age	-0.516	<0.001***	-0.491	<0.001***	-0.546	<0.001***	-0.505	<0.001***	-0.522	<0.001***	-0.513	<0.001***	-0.515	<0.001***	-0.520	<0.001***
Sex (male = 1, female = 2)	0.315	<0.001***	0.253	<0.001***	0.332	<0.001***	0.279	<0.001***	0.335	<0.001***	0.309	<0.001***	0.314	<0.001***	0.268	<0.001***
Facility	-0.244	<0.001***	-0.230	<0.001***	-0.248	<0.001***	-0.258	<0.001***	-0.252	<0.001***	-0.246	<0.001***	-0.245	<0.001***	-0.258	<0.001***
**Main variables**																
BMI			-0.176	0.002**											-0.152	0.006**
Living alone					-0.152	0.005**									-0.142	0.007**
Alcohol intake							-0.132	0.017*							-0.120	0.021*
Short sleep									-0.092	0.096✝					-0.103	0.049*
Smoking											-0.051	0.346				
Lack of exercise													0.004	0.938		
																
R	0.782		0.798		0.796		0.792		0.787		0.784		0.782		0.823	
R^2^	0.612		0.637		0.633		0.628		0.620		0.615		0.612		0.677	
⊿R^2^			0.025		0.021		0.016		0.008		0.003		0.000		0.040	
F	72.563	<0.001***	60.190	<0.001***	59.202	<0.001***	58.034	<0.001***	55.843	<0.001***	54.605	<0.001***	54.032	<0.001***	40.159	<0.001***
⊿F			9.563	0.002**	8.029	0.005**	5.862	0.017*	2.817	0.096✝	0.006	0.346	0.896	0.938	5.516	0.001**

In Step 3, we entered all the control and four significant/marginally significant variables at Step 2. All the main variables, i.e., BMI (R = 0.823, β = -0.152, p = 0.006), living alone (R = 0.823, β = -0.142, p = 0.007), alcohol intake (R = 0.823, β = -0.120, p = 0.021), and short sleep (R = 0.823, β = -0.103, p = 0.049) were significantly associated with GM-BHQ after controlling for sex, age, and facility. These results support H1-H4 but do not support H5-H6. Furthermore, R^2^ 0.677 was significantly higher than the highest R^2^ 0.637 of Step 2 by F-test (p = 0.001). Moreover, R^2^ increment, 0.040, was larger than the “small” level (Cohen, 1977). Therefore, the small but statistically significant differences in R^2^ between Step 2 and Step 3 indicate that GM-BHQ would be negatively associated with unhealthy lifestyles more significantly when they were combined, although its difference may not be large.

## Discussion

Unhealthy lifestyles are bad for human health [1–3, 5]. Similarly, unhealthy lifestyles are bad for brain conditions. Previous studies have indicated that BMI/obesity [7–11], alcohol intake [16–19], short sleep [12–14], smoking [20–22], and lack of exercise [23–25] are negatively associated with GMV. Living alone, which was found to be associated with subjective happiness [30], may also be considered related to GMV. However, to the best of our knowledge, no study has dealt with these six unhealthy lifestyles simultaneously with GMV. If individuals practice these undesirable lifestyles in different magnitudes (i.e. a person who has high alcohol consumption, short sleeping hours, moderate BMI levels, and does live alone), we might be able to find a combination more harmful to GMV compared to the case they were practiced individually.

For that purpose, one hundred and forty-two healthy participants were recruited in two facilities in Japan to acquire their MRI images and answers to the questions composing six lifestyle variables. By our analyses, the six lifestyles were not significantly associated with each other. This means that these behaviors are independently followed by different persons with different extents. For instance, a man who sleeps less is not necessarily obese, or a man who lives alone does not necessarily drink a lot, although we may easily create plausible stories to connect them (e.g., a man who lives alone likes to drown one’s loneliness in alcohol). Four of six unfavorable behaviors, BMI, living alone, alcohol intake, and short sleep, were all associated with GM-BHQ after controlling for age, sex, and facility, not only individually but also when they were entered into a single regression model. Moreover, there were small but significant differences in fit, measured by R^2^, of the models between when these unhealthy variables were entered in an equation at the same time and when they were entered separately, with the former larger than the latter. This indicates that these four unhealthy lifestyle behaviors, which are harmful when looked at individually, are more noxious to GMV when combined, although its difference may not be large. This is a new finding previous research has not explored and might be the greatest contribution to the current literature.

There were several inconsistent results in the current research compared with previous studies. For instance, a previous study found significant reductions in GMV in the left thalamus and amygdala in young adults who have been smoking for less than a decade, indicating that smoking may have a more deleterious effect on brain structure than previously reported [21]. However, we could not find a significant correlation between smoking and GM-BHQ. This is most possibly because the sample did not include a lot of current smokers (the number of current smokers was only 11 out of 142), which may also be due to the recent increased health awareness in Japan. Had we included more smokers in the data, different outcomes might have been obtained.

Another concern is about exercise which turned out to be not associated with GM-BHQ significantly. Although previous research indicated that certain types of exercises and regional GMVs are correlated [23–25], most of these studies were for the elderly, not for healthy middle-aged persons like ours. This suggests that many studies, including ours, have not shown a relationship between exercise and the brain for healthy adults. This suggests that exercise does not have as great an effect on the brain as other variables shown in this study. For example, even for those who exercise frequently, a high BMI from eating a high-calorie diet may have a negative effect on the brain to offset the effects of exercise. Another possible reason for such inconsistency in exercise might be the ambiguous questions used in this study, which only inquired frequency and excluded intensity. For instance, according to the new WHO 2020 guidelines on physical activity, all adults should undertake 150–300 min of moderate-intensity, or 75–150 min of vigorous-intensity physical activity, or some equivalent combination of moderate-intensity and vigorous-intensity aerobic physical activity, per week [41]. Future research may make up for the shortcomings of the current research by using more detailed questions to check for possible associations between certain types of exercise and GMV.

Our research has several limitations. We should not underestimate the differences in nationality. The previous study on GM-BHQ and alcohol consumption [19] had similar results to this study (although different questionnaires were used). It should be noted that both studies used healthy Japanese adults as the cohort or experiments. On the other hand, most GMV-alcohol intake studies held in the west focused on specific populations, such as individuals with alcohol-use disorders [18, 42] or adolescents [16, 17]. This fact indicates that GMV of healthy adults from the west may not be easily influenced by alcohol intake compared to Japanese nationals, whose alcohol metabolisms are distinct from that of Europeans and Africans. This is because the Japanese population’s alcohol metabolism-related single-nucleotide polymorphisms of alcohol dehydrogenase 1B and aldehyde dehydrogenase 2 have been weakening for the past several thousand years [43].

Concern for nationality is also needed for a better interpretation of the result of living alone. Previous studies regarding the association between living alone and health were mostly based on elderly people, with a few exceptions including the research of Raymo [30]. Using the data of the young adult Japanese cohort, Raymo [30] found that those living alone are significantly less happy than those living with others. Further, previous brain research found a positive relationship between the subjective happiness score and regional GMVs [31, 32]. Therefore, our result is consistent with these studies and at the same time bridges them by newly finding a direct connection between living alone and lower GM-BHQ in healthy adults. However, as these studies and ours are commonly based on the Japanese cohort, we may have to carefully interpret the results from the viewpoint of generalizability. For example, these linkages may perhaps be due to the Asian collectivistic culture that emphasizes harmony with others, wherein people who easily or publicly express negative feelings are often regarded as immature and weak [44]. Indeed, using the opinions from 575 undergraduates, which were obtained by vignette-based experiments, it was reported that negative biases toward depression were stronger in the Japanese collectivistic context than in the US individualistic context [45]. Under such circumstances, Japanese people may consciously or subconsciously need a companion who can listen to their complaints more than western people do because Japanese individuals are less likely to display negative emotions outside the home and thus have more need for a companion inside the home. Furthermore, future research could elaborate on what “living alone” really indicates. We associated it with lower happiness following Raymo [30]. However, living alone may accompany various meanings. For instance, in a previous study using data from 16,849 adults, social isolation was a predictor of mortality on par with smoking, obesity, elevated blood pressure, and high cholesterol [45], which indicates that isolated people may have less access to tangible resources and knowledge that help promote better health [46]. Similarly, it is reported that students living alone tend to make relatively worse food choices, such as eating irregularly, skipping meals often, and relying more on the foods outside [47]. Therefore living alone leads to poor nutrition for students [48]. This may be caused by the absence of family members or companions who can help take care of their health. Therefore, by carefully examining the elements of living alone in future research, we may consider a more constructive solution to prevent brain atrophy.

We used a measure of whole-brain normalized gray matter volume. However, many researchers argue that whole-brain volume is not an informative measure if there is no detail about regional brain volume because certain brain areas are more likely to be affected by particular lifestyle factors. Similarly, as the results of a study will be influenced by the questionnaire used, the results of this paper should be verified by different questionnaires in the future.

Although the use of multiple facilities is a strength for the generalizability of the study, it can also be a limitation. This limitation was addressed by the steps taken to control the facility. This procedure is rational as whole-brain volume differs between facilities but that would not interfere with the pattern of findings, as it might if the authors were to evaluate specific regions [37, 38]. If there was any evaluation of specific regions of the brain, more measures would need to be taken to account for this facility difference.

Lastly, this study is cross-sectional and the findings in a strict sense report only correlations of unhealthy styles with GMV, not cause and effect chains.
